# First‐in‐human microelectrode recordings from the vagus nerve during clinical vagus nerve stimulation

**DOI:** 10.1002/epi4.13083

**Published:** 2024-10-28

**Authors:** Mikaela Patros, David G. S. Farmer, Kegan Moneghetti, Matteo M. Ottaviani, Shobi Sivathamboo, Hugh D. Simpson, Terence J. O'Brien, Vaughan G. Macefield

**Affiliations:** ^1^ Department of Neuroscience, School of Translational Medicine, The Alfred Centre Monash University Melbourne Victoria Australia; ^2^ Baker Heart and Diabetes Institute Melbourne Victoria Australia; ^3^ Department of Neurosurgery Università Politecnica Delle Marche, Ospedali Riuniti Torrette di Ancona Ancona Italy; ^4^ Department of Neurology Alfred Health Melbourne Victoria Australia; ^5^ Department of Medicine and Neurology The Royal Melbourne Hospital Parkville Victoria Australia

**Keywords:** epilepsy, microelectrode, nerve fibers, vagus nerve stimulation, VNS

## Abstract

**Introduction:**

Vagus nerve stimulation (VNS) is an effective treatment for people with drug‐resistant epilepsy. However, its mechanisms of action are poorly understood, including which nerve fibers are activated in humans during VNS in typical clinical settings and which are required for clinical efficacy. In particular, there have been no intraneural recordings of vagus nerve fiber activation in awake humans undergoing chronic VNS. In this study, for the first time, we report recordings from the vagus nerve in this setting.

**Methods:**

The recordings were performed using a sterile tungsten microelectrode inserted percutaneously into the cervical vagus nerve under ultrasound guidance. The clinical VNS systems were used to deliver stimulation while activity in the vagus nerve was recorded.

**Results:**

In addition to activating myelinated axons at low currents, we provide evidence that VNS can also activate unmyelinated C fibers in the vagus nerve at currents <1 mA.

**Conclusions:**

These results add to our understanding of how VNS exerts its beneficial effects in drug‐resistant epilepsy.

**Plain Language Statement:**

Here we describe for the first time, electrical recordings from the vagus nerve in awake drug‐resistant epilepsy patients with an implanted vagus nerve stimulation (VNS) device. We found that the VNS device was able to activate both myelinated and unmyelinated fibers within the vagus nerve, which contributes to our understanding of how VNS works in the context of drug‐resistant epilepsy.

**Key points**

- While vagus nerve stimulation (VNS) can reduce seizure burden in drug‐resistant epilepsy (DRE), it is not known which vagal nerve fibres VNS excites.
- Previous studies were conducted on surgically exposed vagus nerves in anaesthetized humans.
- Here we used a tungsten microelectrode inserted under ultrasound guidance into the cervical vagus nerve in two awake participants with DRE.
- Our preliminary results indicate that VNS can excite unmyelinated as well as myelinated axons at currents <1 mA.
- This information will help determine the optimal stimulation parameters for treating DRE while limiting side‐effects.

## 1 INTRODUCTION

Drug‐resistant epilepsy (DRE)—the failure to attain seizure freedom after two or more adequately trialed antiseizure medications (ASMs)^1^—represents a large and significant treatment gap in the field. For people with DRE, neuromodulation is an effective treatment option, and vagus nerve stimulation (VNS) is one of the most established forms of neuromodulation.^2^ VNS involves chronic intermittent stimulation of the left vagus nerve using an implanted device. The device is made up of two components: the bipolar cuff electrodes, which are wrapped around the left vagus nerve, and the generator, which is implanted subcutaneously below the left clavicle in the anterior wall of the chest.^3^

The human cervical vagus nerve is composed of myelinated axons (A and B fibers) and unmyelinated axons (C fibers), which have differing thresholds for activation.^4^ There is also a subtype of A fibers known as A‐delta fibers which may also be implicated in the sensory activation pathway contributing to the antiepileptic effects of VNS, but this has not been systematically investigated in humans. Due to their biophysical properties, C fibers require higher currents to reach the threshold, and it has been argued that these currents could not be achieved during clinical VNS in the treatment of DRE.^5^ It is clear that better characterization of fiber activation by VNS in humans will help us understand the mechanisms of VNS and allow for optimization of stimulation parameters. Despite this, there have been few studies of fiber activation in humans using typical clinical VNS systems.

While parameter ranges that are generally effective are well established, there is no consensus on the optimal stimulation parameters, given (1) the lack of systematic studies on optimal parameters and (2) absence of reliable biomarkers to assess the short‐term responses to VNS and thereby tailor stimulation to the individual.

Here we report a case series that describes the first microelectrode recordings from the cervical vagus nerve in two awake DRE patients with chronically implanted VNS systems. Using the minimally invasive, ultrasound‐guided approach that we developed for recording from the human vagus nerve,^6^ our aim in this pilot study was to determine the output currents needed to recruit vagal fibers using a clinical VNS device.

## 2 METHODS

This case series was performed under Alfred Health Human Research Ethics Committee approval 341/22 and satisfied the principles of the Declaration of Helsinki.

Two male participants in their early 30s (Table 1) with DRE receiving VNS were recruited from Alfred Health outpatient clinics in 2023.

**TABLE 1:** Patient demographics.

Patient ID	Time since VNS implant	VNS Implanted	Stimulation parameters	Epilepsy syndrome	Device	Anti‐seizure medications	Other medications
P1	4 years 5 months	Oct‐19	2.0 mA, 20 Hz, 250 μs, ON 7 s, OFF 0.5 min, duty cycle 30%	Focal	AspireSR M106	Lacosamide 200 mg BD, zonisamide 500 OD, clobazam 20 mg BD	Rosuvastatin 10 mg OD
P2	2 years 1 month	May‐21	1.75 mA, 20 Hz, 500 μs, ON 7 s, OFF 0.2 min, duty cycle 58%	Focal	SenTiva M1000	Carbamazepine 500 mg BD, clobazam 10 mg OD, clonazepam 0.5 mg BD, lamotrigine 200 mg BD, topiramate 200 mg BD	

*Note*: Time since vagus nerve stimulator (VNS) implant was calculated from the implant date to the date of the patient visit for the vagus recordings. Stimulation parameters as recorded on the day are listed as well as the model of each VNS device. Epilepsy details include epilepsy syndrome (focal), antiseizure medications and dosing frequency—once daily (OD), twice daily (BD)—and any other medications patients were taking concurrently.

Participants underwent continuous noninvasive blood pressure monitoring via finger plethysmography (NOVA, Finapres Medical Systems, Enschede, The Netherlands), sampled at 400 Hz. Additionally, respiration was monitored using a piezoelectric transducer around the chest (ADInstruments, Sydney, Australia), sampled at 100 Hz, and the electrocardiogram (ECG) was recorded with surface electrodes on the chest (BioAmplifier; ADInstruments), sampled at 2 kHz.

A clinical ultrasound scan of the neck was performed to measure the intima‐media thickness (IMT) of the left carotid artery to determine the presence of plaque. An IMT value of above 1.00 mm resulted in exclusion from the study. The position of the VNS cuff electrodes on the vagus nerve was then determined under ultrasound guidance. This aided in the identification of the optimal insertion point of the recording microelectrode, approximately 10–20 mm proximal to the cathode (cephalad electrode). With the head rotated contralaterally, a sterile tungsten microelectrode (Frederick Haer, Bowdoin, ME, USA, diameter 200 μm, length 75 mm) was inserted manually through the skin overlying the anterior border of the sternocleidomastoid muscle under ultrasound guidance. The microelectrode was advanced in a dorsolateral trajectory towards the vagus nerve until we confirmed on the ultrasound that the microelectrode tip had penetrated the nerve sheath. Once the microelectrode tip was situated within a fascicle of the nerve, the participant's own VNS device was used to stimulate the nerve, and the parameters were adjusted using a telemetric wand. Action potentials recorded via the microelectrode were amplified (gain 2 × 10^4^, bandpass 0.3–5.0 kHz) with a low‐noise headstage (NeuroAmpEX, ADInstruments) and stored (20 kHz sampling) via a computer‐based data acquisition system (PowerLab 16/35 and LabChart 7 for Mac; ADInstruments). The VNS was set at a frequency of 1 Hz and pulse width of 130 μs. The current output was gradually increased from 0 mA to their maximal clinical stimulation current in 0.125 mA increments to identify recruitment of vagal fibers. Five stimulation cycles were recorded at each current step.

## 3 RESULTS

**FIGURE 1:**
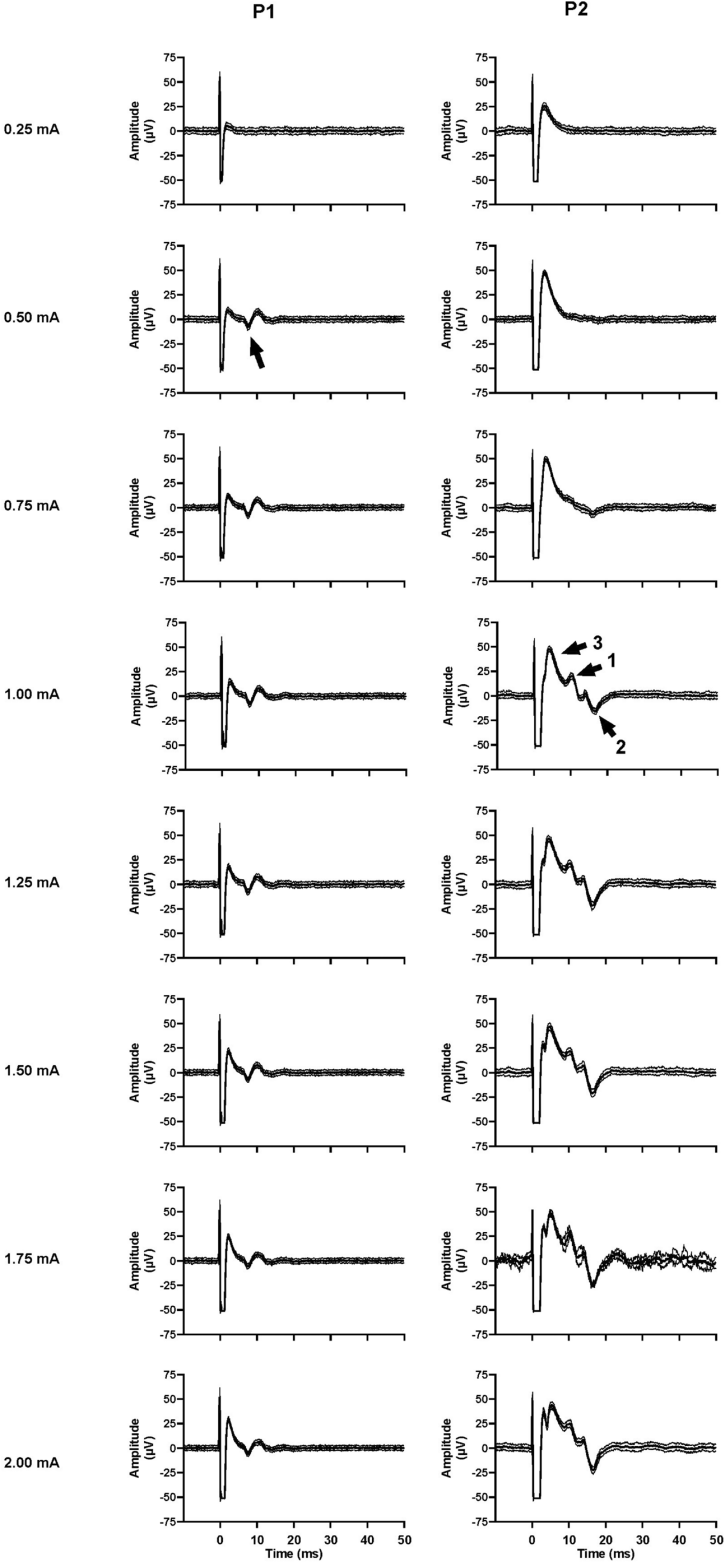
Event‐triggered averages of compound evoked potentials recorded from the cervical vagus nerve in two epilepsy patients with implanted VNS devices. The stimulus artifact was used as the trigger to generate averages of nerve activity and occurs at time 0; current intensity is displayed on the left. Pulse width was 130 μs. Each panel under P1 shows a mean ± SD of 40 stimuli, while each panel under P2 shows the mean ± SD of 30 stimuli. Arrows indicate evoked action potentials.

Both participants tolerated the procedure well, with only minimal discomfort reported. There were no complications such as bleeding, infection, or inadvertent vascular puncture. As the current was increased from 0.125 to 0.375 mA in P1, a single negative‐going deflection was observed with a fixed peak latency of 7 ms (Figure 1). With an estimated conduction segment of ~10 mm the conduction velocity of this negative‐going potential was estimated to be ~1.4 m/s, that is, within the C‐fiber range.

For P2, there were no compound action potentials detected with stimulus currents of 0.25–0.75 mA (Figure 1). At 1 mA, an early deflection could be seen after the stimulus artifact, with two distinct compound action potentials being observed at peak latencies of 10 and 16 ms, respectively. Based on the ~20 mm conduction segment between the stimulating cathode and recording microelectrode the conduction velocities for these two components were calculated to be ~2.0 and 1.25 m/s, respectively. The latter component was negative‐going, indicating that this potential was generated by unmyelinated axons (C fibers). As stimulus intensity increased, the earliest component—positive‐going spikes generated by myelinated axons^7^—occurring at a peak latency of 3 ms (~6.7 m/s) became more readily defined, yet there were no changes in the two later components up to 2 mA.

## 4 DISCUSSION

Here we report the first microelectrode recordings from the cervical vagus of people with epilepsy treated with chronically implanted VNS devices. Direct recordings from the vagus nerve in epilepsy patients provide the opportunity to understand fiber activation at various stimulation settings and to identify which currents are required to activate specific fiber types.

Previously, there have been investigations of the electrophysiology of the surgically exposed vagus nerve.^8^, ^9^ One study examined the effects of varying stimulation parameters on vagal fiber activation in DRE patients aged between 4 and 31 years old.^9^ The authors of this paper found that the vagus nerve in children required a higher current to reach threshold activation compared to adults, with conduction velocity increasing with age. For those older than 12 years, conduction velocities ranged from 8.8 to 12.6 m/s, with a mean of 10.2 ± 1.2 m/s. One limitation of this investigation is that researchers were not stimulating the vagus nerve with a clinical VNS device but using stimulating forceps with a much smaller surface diameter and hence a higher current density at the points of contact. This is not comparable to the clinical VNS device as the helical cuff electrodes cover a much larger area of the vagus nerve and current travels along the nerve from the cathode to anode, meaning it may not penetrate to deep fascicles. In addition, the recordings by Koo et al.^9^ were performed intraoperatively and under anesthesia, conditions that are less comparable to the chronic stimulation settings we recorded under.

Two previous studies have used hook electrodes under the surgically exposed vagus nerve to record compound action potentials evoked by VNS, identifying three peaks of compound action potentials: fast‐conducting A fibers and slower‐conducting Aδ‐ and C fibers.^4^, ^5^ Using intraneural microelectrodes in the current study, we could also detect slowly conducting, negative‐going potentials that are characteristic of C fibers.

Our investigations show activation of C fibers in two patients at low stimulation intensities. The importance of studying axonal activation in patients with VNS is highlighted here, given there is much debate as to whether C fibers are responsible for any of the anticonvulsant effects of clinical VNS.^8^, ^10^ It has been shown that C fibers require higher currents for activation,^8^ so our finding of C‐fiber activation at lower currents is significant.

A limitation of our study was not performing a longitudinal ultrasound scan to measure the conduction distance between the stimulating cathode and recording microelectrode, as opposed to measuring from the surface of the neck. Thus, the conduction distance was approximated based on an estimated distance between the position of the recording microelectrode and stimulating cathode. Future recordings will include precise measurements of the conduction segment. Additionally, we note that the recordings we present are focal (i.e., they sample evoked action potentials from a small area within only a single fascicle of the nerve). As such, any interpretation should not exclude the certainty that VNS is activating fascicles containing myelinated fibers not recorded here by the microelectrode tip.

In conclusion, we present the first vagus nerve recordings in conscious people with epilepsy who have an implanted VNS device. Our preliminary investigations have identified the activation of myelinated and unmyelinated axons. Future studies will help define the stimulus characteristics required to activate different fiber classes during VNS, including whether deeper fascicles within the nerve receive effective stimulation.

## CONFLICT OF INTEREST STATEMENT

MP, DGSF, KM, and MMO report no disclosures. SS is the recipient of a National Health and Medical Research Council (NHMRC) Investigator Award (2025610). She is supported by Research Program Grants from the National Institute of Health (NIH) (1U54AT012307‐01 and 1R01NS123928‐01). She reports salary support paid to her institution from Jazz Pharmaceuticals for clinical trial‐related activities; she receives no personal income for these activities. HDS has received travel support from LivaNova for educational purposes and has received competitive grant funding from the Medical Research Future Fund (MRFF) in Australia. TOB's institution has received research funding from Eisai, UCB Pharma, LivaNova, ES Therapeutics, and Kinoxis Therapeutics. He has also received competitive grant funding from the NHMRC, MRFF, NINDS, and the DoD. VGM receives funding from the NHMRC and the NIH. We confirm that we have read the Journal's position on issues involved in ethical publication and affirm that this report is consistent with those guidelines.

## Data Availability

Data are available upon reasonable request.
